# Effects of Aerobic Exercises on Serum Levels of Myonectin and Insulin Resistance in Obese and Overweight Women

**DOI:** 10.25122/jml-2018-0033

**Published:** 2018

**Authors:** Mohammad Pourranjbar, Najmeh Arabnejad, Khatereh Naderipour, Forouzan Rafie

**Affiliations:** 1. Neuroscience Research Centre, Kerman University of Medical Sciences, Kerman, Iran; 2. Kerman University of Medical Sciences, Kerman, Iran; 3. Sport Physiology Department, Sarcheshmeh, Kerman, Iran

**Keywords:** Myonectin, insulin resistance, obese women, exercise

## Abstract

**Background and Aim:** Obesity is associated with cardiovascular diseases, metabolic syndrome, and diabetes and insulin resistance. Myonectin is a myokine mostly secreted from skeletal muscles and inversely associated with obesity. The aim of the present study was to evaluate the effects of 8 weeks of aerobic exercises on serum levels of myonectin and insulin resistance in obese and overweight women.

**Materials and Methods:** Eighty obese women were assigned to exercise (34) and control groups (46). The exercise program comprised three weekly 45-minute sessions of aerobic exercise training for 8 weeks that included running with 50–70% of maximum heart rate (first 2 weeks – 50%; second week – 60%; third week – 65%; and the last 2 weeks by 70% of maximum heart rate). Twenty-four hours before and after the training session, fasting myonectin serum levels were measured. ANCOVA was used to assess differences between the groups.

**Results:** Serum levels of myonectin in the experimental group increased significantly (P=0.000); however, insulin resistance significantly decreased in the experimental group (P=0.000).

**Conclusion:** Therefore, considering the role of myonectin in increasing fatty acid uptake, exercise training can play an essential role in decreasing obesity-related diseases and metabolic syndrome; this effect is partly related to the roles of myonectin. Therefore, the use of this type of exercise is recommended to reduce the risk of diseases associated with obesity and metabolic syndrome.

## Introduction

Obesity is characterized by the growth of adipose tissue due to the increase in the number and size of adipose tissue cells. Obesity and the resulting physiological changes can be considered a potential risk factor for metabolic syndrome, type 2 diabetes, cardiovascular diseases, cancer and other conditions [1,2]. Diabetes is a metabolic disease characterized by insulin resistance in the target tissue and a chronic increase in blood glucose [3]. The relationship between obesity and insulin resistance has been reported in previous studies, but the mechanisms associated with insulin resistance due to obesity are not well defined [4]. Adipokines and myonectines may also play an important role in the development of diseases associated with obesity and insulin resistance [2].

The CTRP15 myonectin is a newly discovered mycophenolate, often released by skeletal muscles and as a free protein involved in metabolic functions [5,6]. It has been shown that Myonectin levels have an inverse relationship with obesity, as well as Myonectin reduces the amount of free fatty acid in the circulation, which is done by absorbing more acid from the tissues [5]. Laboratory studies have shown that elevated intracellular levels of calcium after exercise often increase the expression of myonectin in skeletal muscles [6,7]. Many studies have shown that myonectin ameliorates metabolic health outcomes by decreasing circulatory levels of free fatty acids and by increasing uptake in adipose and liver tissues [5]. Obesity decreases the levels of myonectin [5] and exercises increase the expression of the myonectin gene [5,8,9].

Lim et al. (2012) showed that 10 weeks of moderate-intensity training led to a significant reduction in the amount of myonectin and insulin resistance in older and younger patients; in this study, there were changes in myonectin levels with adiponectin changes and maximum oxygen intake, with an inverse relationship between insulin resistance indexes [12]. In the study by Seldin et al. in 2012, the increase in myonectin expression in muscle and circulation was observed after two weeks of aerobic exercises [5]. Kraniou et al. showed that endurance training improves insulin sensitivity in young, middle-aged and insulin-resistant subjects, which is attributed to concurrent weight loss and positive regulation of protein expression and skeletal muscle glucose [13]. In another study, Peterson showed that nine weekly treadmill training exercise sessions increased myonectin gene expression in obese male rats and reduced the expression of myonectin genes by exercise in both groups of lean and obese mice, while the concentration of myonectin protein after exercise increased [14]. In a study by Bagarabadi et al. (2012), 12 weeks of regular aerobic exercises resulted in a significant decrease in insulin resistance in type 2 diabetic patients [15]. In reviewing the studies on the effects of exercise on myonectin and insulin resistance, contradictory results were observed; in some cases, myonectin and insulin resistance increased [6] and in some others, they decreased [14–17]. As reported in the above studies, the effects of exercise on Myonectin are still unknown; therefore, in this study, we aimed to determine the effects of aerobic exercise training on myonectin levels and insulin resistance in obese and overweight women.

## Methodology

First, 100 obese and overweight women completed the Medical Assessment Questionnaire (Health Assessment) and demographic data questionnaire including age, gender, weight, height, and body mass index (BMI). Finally, based on inclusion and exclusion criteria, 80 healthy subjects with an age range of 35–45 years and (BMI>25 kg/m2) were selected by cluster sampling from different regions of Kerman. Subjects who had a physical problem in motor skills for performing exercise programs, metabolic illness and those who had previously been diagnosed with type 2 diabetes mellitus or glucose intolerance or drug use and active participants (athletes) were excluded from the study. For ethical considerations, all the participants were fully informed of experimental procedures before signing a written informed consent; all the stages of the study were explained to the subjects, and then written consent was collected from the participants.

The subjects were matched regarding height, weight, body mass index, fasting glucose levels, oral-glucose-tolerance-test response and waist circumference, and randomly divided into experimental (n=38) (mean age of 38.15±2.33 years) and control (n=42) (mean age of 38.89±1.78 years) groups. Since myonectin can be influenced by nutrients, we also controlled the diet in the samples by giving a similar diet in during the study period.

The exercise program consisted of 24 aerobic exercise sessions for 8 weeks, three sessions in a week (10 minutes of warm-up training, 30 minutes of running with 50–70% of maximum heart rate and 5 minutes of cooling down), with the first two weeks exercising at 50% of maximum heart rate, in the second two weeks at 60%, in the third two weeks at 65%, and in the last two weeks at 70% of the maximum heart rate. To control exercise intensity, a pacemaker and a polar clock were attached to the subjects, and a range was defined for the hour; In order to control the intensity of the training, the Polar heart rate monitor armband was closed to the participants and adjusted for one hour. If the intensity of the exercise reaches less or more than the range, the clock warned with a beep.

Twenty-four hours before the first training session and 24 hours after the last session, venous blood samples were taken from the subjects. The blood samples were then centrifuged at 3000 rpm for 10 minutes. All the samples were stored in a refrigerator at −80°C for biochemical analysis.

Serum myonectin and insulin concentrations were measured by Abcam company kits (SK00393-09). Serum glucose concentrations by using an enzymatic colorimetric method (based on reaction glucose oxidase) with kits made by Pars Tests Company was measured. Insulin resistance was also calculated using the following formula (Insulin resistance = plasma insulin × plasma glucose: 22.50).

The current study was approved by the Ethics Committee of the Kerman University of Medical Sciences.

### Statistical Analysis

Descriptive statistics were used to describe data (means and standard deviations). After examining the normal distribution of data with the Kolmogorov-Smirnov test, ANCOVA was used (at a significant level of P<0.05) to examine the differences between the two groups. Data were analyzed with SPSS 21.

## Results

Eighty obese and overweight women (with an age range of 35–45 years) were included in this study. The subjects were matched for age, height, weight and BMI, and divided into one case and one control group. The normal distribution of data was verified using the Kolmogorov-Smirnov test and homogeneity of variances by using Levene’s test (P≥0.05). Individual characteristics, including height, weight, body mass index (P=0.631), and physiological parameters (myonectin) (P=0.755) and insulin resistance (P=0.565) of the subjects are shown in Table 1. There were no significant differences between the groups at baseline (Table 1).

**Table 1:** Anthropometric characteristics and measured indices (standard deviation ± mean)

Groups		Height Cm	Weight (Kg)	BMI (kg/m^2^)	insulin resistance	Myonectin (ng/ml)
Exercise	Before	160.45±5.50	9.83±78.85	2.33±30.07	0.11±3.52	0.10±0.24
	After	5.43±160.42	8.67±74.02	2.27±28.21	0.09±2.33	0.17±0.39
Control	Before	5.3±160.20	9.67±79.80	2.70±30.01	1.3±3.88	0.44±0.22
	After	5.4±160.20	7.89±79.15	2.16±30.07	1.2±3.87	0.15±0.23

Paired t-test showed that the myonectin level, insulin resistance and BMI were significantly different in the experimental group before and after aerobic exercise, (P<0.05); on the other hand, in the control group no significant difference was observed at the baseline and after 8 weeks (P>0.05) (Table 2).

**Table 2:** Paired T–Test (before and after exercise in each group)

Variables groups/time		Mean	Std. Deviation	95% Confidence Interval of the Difference		t	df	Sig(2tailed)
				Lower	Upper			
insulin resistance	Exercise (Before – After)	–1.156	0.176	–1.195	–1.116	–58.495	78.000	*0.000
insulin resistance	Control (Before – After)	–0.076	2.119	–0.550	0.399	–0.318	78.000	0.751
Myonectin	Exercise (Before – After)	0.188	0.444	–0.287	–0.089	–3.768	78.000	*0.000
Myonectin	Control (Before –After)	0.043	0.208	–0.003	0.090	1.851	78.000	0.068
BMI	Exercise (Before – After)	–1.476	2.777	–2.090	–0.862	–4.783	80.000	*0.000
BMI	Control (Before –After)	0.604	2.714	–0.004	1.212	1.979	78.000	0.071

* Significant at P<0.05

Independent t-test showed that the serum myonectin level increased significantly in the experimental group compared to the control group (P=0.000); on the other hand, the serum insulin resistance level in the experimental group was significantly lower than that in the control group (P=0.000) (Table 3).

**Table 3:** Independent Samples T-Test (between control and exercise groups)

Variables	Mean Difference	Std. Error Difference	95% Confidence Interval of the Difference		df	t	Sig. (2-tailed)
			lower	upper			
Myonectin	.1528025	.0267070	.2055540	.1000511	157	5.721	*.001
insulin resistance	–.4668578	.1600400	.7829671	–.1507485	157	2.917	*.004
BMI	.5976619	.3240124	.0418716	1.2376476	157	1.846	*.027

* Significant at P<0.05

## Discussion

Overweightness and obesity have increased significantly over the past few years [18]. Previous studies have shown that both the absolute body fat and the central distribution of lipids, including abdominal visceral fat, are closely associated with diabetes, hypertension, increased blood lipids and cardiovascular diseases [19]. Obesity with excessive accumulation of visceral fat around and within the abdominal organs and increased flow of fatty acids to the liver disrupts insulin secretion, increasing insulin resistance and production of glucose in the liver [20].

According to the current results, there were significant differences in mynoectin levels and insulin resistance in the aerobic exercise group compared to the control group after 8 weeks.

Myonectin is a novel myokine agent secreted by skeletal muscles; it affects fat metabolism and decreases the level of circulating lipids. Expression of myonectin occurs in obesity and when there is an intake of additional calories [5]. The findings were consistent with those reported by Seldin et al. (2012), who showed that obesity has an inverse relation with the amount of plasma myonectin [5]. The increases in myonectin levels in the exercise group compared with the control group in the current study can be attributed to the decrease in BMI. In previous studies, the circulating levels of myonectin decreased with obesity by reducing the amount of free fatty acids and absorbing more acids from the tissues [5]. In addition, myonectin contributes to the phosphorylation of adenosine monophosphate kinase, the increase in glucose uptake and the oxidation of fatty acids [21]. In this work, endurance training significantly increased the circulating levels of myonectin in obese women, which was anticipated based on the literature. The expression of the myonectin gene would decrease by obesity and would increase by exercise [8]. Seldin et al. (2012) showed that voluntary exercise by wheel running for three weeks increased the expression of the myonectin gene [5]. It can be claimed that exercise training, probably by increasing myonectin levels in obese individuals, leads to better absorption of glucose and lipids in the body and ultimately helps control and modify lipid profiles and insulin sensitivity in obese and overweight people. Contrary to our results, Peterson et al. (2014) found that treadmill running for 9 weeks reduced myonectin expression, regardless of obesity status; however, in the Paterson study, the serum levels of myonectin were not considered [14].

Moreover, the results of this survey demonstrated that insulin resistance and serum insulin levels decreased significantly in obese women after a period of aerobic training, demonstrating the positive effects of aerobic exercise on improving insulin-dependent indices in obesity. In this respect, the results of a study by Fox et al. showed that even a session of endurance training could improve insulin sensitivity in obese individuals and optimize insulin function for two days [22]. Likewise, in another work, Le et al. (2016) examined the effects of exercise intervention on insulin resistance in overweight/obese women and showed a decrease in insulin resistance in the central body region among overweight and obese women after 8 months of aerobic exercise [23]. On the contrary, Faramarzi et al. (2016) examined the effects of 12 weeks of rhythmic aerobic exercise with core stability training on serum insulin levels and insulin resistance in overweight women and reported that this kind of training had no significant effect on insulin levels and insulin resistance in overweight women [24].

To explain our results, it can be stated that the proinflammatory/stress pathway plays an important role in insulin resistance in obese and diabetic patients; aerobic exercises appear to increase the distribution of fatty acids and reduce their accumulation by preventing the activation of inflammatory pathways and their negative effects on signaling and insulin sensitivity [25]. Also, in terms of molecular effect, insulin-induced glucose absorption decreases in skeletal muscles of insulin-resistant individuals due to the decrease in the transport of glucose-4 carrier protein to the plasma membrane. Changes in carrier protein after exercise have been reported, which is mainly regulated by the activation of adenosine monophosphate kinase [26]. Furthermore, muscle contraction increases the permeability of the membrane to glucose, possibly due to an increase in the number of glucose transporters in the plasma membrane (Glut-4). Thus, after exercise, an increase in the amount of glut-4 in the trained muscle improves the action of insulin in the metabolism of glucose [10]. Mann et al. (2014), in a review article on insulin sensitivity changes in response to exercise training, showed that the positive effects of aerobic training on the glycemic index and insulin sensitivity could be used as a suitable model for control and improvement of insulin markers [27]. Ginghina et al., in their study on the modern risk stratification in coronary heart disease, showed that one of the most important factors in the development of heart disease is impulsivity and lack of mobility [28]. On the other hand, Firouzabadi et al. showed the effect of physical activity on the life quality of coronary artery bypass graft patients [29]. Since obesity and subsequent physical and mental problems [30] are increasing in today’s modern world, exercise and physical activity are recommended to reduce obesity and weight gain, especially in women [31].

One of the limitations of the current study is that we did not consider a diet history questionnaire. In addition, we only considered the immediate effects of exercise on myonectin and insulin resistance levels after training and the changes during the study were not assessed, an action suggested for further studies. Besides, in this study, only female samples were selected; for a wider generalization of the results, a study on both sexes is recommended. Also, a comprehensive study is suggested to consider multiple and related physiological factors in obese people. Further research should be conducted into molecular and histopathological mechanisms of expression and secretion of myonectin and insulin resistance.

## Conclusion

The results of this study showed that engaging in eight weeks of aerobic training resulted in a significant reduction in insulin resistance and a significant increase in myonectin in obese women. Hence, exercise can be used to control and improve the risk factors of insulin resistance such as diabetes and cardiovascular disease and should be encouraged in obese and overweight individuals.

## Acknowledgments

The authors would like to acknowledge the subjects for their participation.

## Conflict of Interest

The authors confirm that there are no conflicts of interest.

## Ethical approval

All procedures performed in studies involving human participants were in accordance with the ethical standards of the institutional and/or National Research Committee and with the 1964 Helsinki Declaration and was evaluated and approved by the Ethical Committee of Kerman University of Medical Sciences (EC: K/96/59).

## Informed consent

Informed consent was obtained from all individual participants included in the study.
